# Profilin Isoforms in Health and Disease – All the Same but Different

**DOI:** 10.3389/fcell.2021.681122

**Published:** 2021-08-12

**Authors:** Kai Murk, Marta Ornaghi, Juliane Schiweck

**Affiliations:** Institute of Biochemistry, Charité Universitätsmedizin Berlin, Berlin, Germany

**Keywords:** profilin, actin, cytoskeleton, central nervous system, amyotrophic lateral sclerosis, Fragile X syndrome, Huntington’s disease, spinal muscular atrophy

## Abstract

Profilins are small actin binding proteins, which are structurally conserved throughout evolution. They are probably best known to promote and direct actin polymerization. However, they also participate in numerous cell biological processes beyond the roles typically ascribed to the actin cytoskeleton. Moreover, most complex organisms express several profilin isoforms. Their cellular functions are far from being understood, whereas a growing number of publications indicate that profilin isoforms are involved in the pathogenesis of various diseases. In this review, we will provide an overview of the profilin family and “typical” profilin properties including the control of actin dynamics. We will then discuss the profilin isoforms of higher animals in detail. In terms of cellular functions, we will focus on the role of Profilin 1 (PFN1) and Profilin 2a (PFN2a), which are co-expressed in the central nervous system. Finally, we will discuss recent findings that link PFN1 and PFN2a to neurological diseases, such as amyotrophic lateral sclerosis (ALS), Fragile X syndrome (FXS), Huntington’s disease and spinal muscular atrophy (SMA).

## Introduction

Over forty years ago, the first profilin was identified as a small actin monomer binding protein with the ability to inhibit actin polymerization *in vitro* (Carlsson et al., 1977). From there, the profilin field has expanded enormously: Originally described as actin sequestering protein, a wealth of literature established profilin as driving and directing force for actin polymerization and network homeostasis. Besides canonical actin dynamics, miscellaneous profilin ligands and numerous functional studies have linked profilin to diverse fields of cell biology including membrane trafficking, signaling, synaptic scaffolding, nuclear export, mRNA splicing, and transcription (see Supplementary Table 1). The (co-)expression of different profilin isoforms generates another level of complexity and raises the question as to whether these proteins are functionally unique or if they are redundant. Moreover, recent studies show the association of profilin isoforms with the onset and/or progression of several diseases through either mutation in their ligands or in the profilin isoforms themselves. In this review article, we begin by summarizing main aspects of profilin properties and functions. We introduce profilins with their typical structural and biochemical features including their function as actin regulators. Furthermore, we will discuss the functional diversity of profilin isoforms of higher animals in cellular processes. We will then focus on overlapping and unique roles of PFN1 and PFN2a in the central nervous system (CNS). Finally, we will give insight into the biomedical relevance of these profilin isoforms through their emerging roles in neurological diseases.

## Profilin Genes, Structure and Biochemical Properties

**FIGURE 1:**
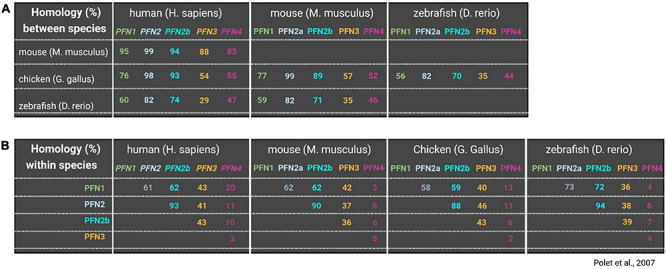
Comparison of profilin isoform amino acid sequences between and within organisms. Upper panel **(A)** shows homologies (%) of all profilins between different species including human, mouse, chicken and zebrafish. Lower panel **(B)** shows homology (%) of different profilins within the same species (lower panel). Blank squares were left blank to avoid redundant information. Information on profilin homologies are based on Polet et al. (2007).

The identification of 172 paralogous and orthologous profilin genes in lower eukaryotes, fungi, plants and animals supports the conservation of profilins throughout evolution (Pandey and Chaudhary, 2017). Even cyanobacterial and viral profilin genes exist, which likely were hijacked from eukaryotic cells by horizontal gene transfer (Blasco et al., 1991; Guljamow et al., 2007). Most lower eukaryotes usually have one profilin gene (Pandey and Chaudhary, 2017). Exceptions to the rule are the free-living amoeba *Dictyostelium discoideum* and the slime mold *Physarum polycephalum*, which express, either constitutively or temporally, additional profilin isoforms (Binette et al., 1990; Arasada et al., 2007). Most studied multicellular organisms express several profilin isoforms some of which are generated by alternative splicing and/or are expressed in a tissue-specific manner. *In silico* analyses of profilin amino acid sequences from various origins show degrees of homologies down to less than 25% between profilins from lower eukaryotes and animals (Figure 1A). But also profilin isoforms in the same organism may substantially differ in their amino acid composition (Figure 1B; Polet et al., 2007). In contrast to this variability in amino acid sequences, the secondary and tertiary structures of profilins are conserved. Seven beta-strands form a compact core surrounded by four alpha-helices. N- and C-termini of profilins are part of alpha-helices and are positioned adjacent to each other (Cedergren-Zeppezauer et al., 1994; Mahoney et al., 1997; Haikarainen et al., 2009). A typical profilin protein contains functional binding sites for G-actin, poly-proline-motifs, and phosphoinositides: the G-actin binding site of profilin occupies a relatively large surface area involving residues of the beta-strands 4,5 and 6, the alpha-helices 3 and 4 as well as interspacing loop regions (Schutt et al., 1993). The interaction with profilin induces a conformational change in G-actin toward a wider nucleotide binding pocket and thereby largely accelerates the nucleotide exchange from ADP to ATP (Perelroizen et al., 1996; Selden et al., 1999). In addition, profilin uses the actin binding site to interact with two other ligands: the actin related protein 2 (Arp2), a subunit of the Arp2/3 complex (Mullins et al., 1998), and gephyrin, which is involved in cellular metabolism through its role in molybdenum cofactor synthesis, but also serves as a postsynaptic scaffolding protein at inhibitory synapses in neurons (Stallmeyer et al., 1999; Giesemann et al., 2003). On the opposite side of the profilin molecule, aromatic amino acids in N- and C-terminal areas form the binding groove for poly-proline stretches (Björkegren et al., 1993; Haarer et al., 1993; Kaiser and Pollard, 1996). Typical poly-proline motifs for profilin binding are continuous sequences of five to ten prolines interspaced by single glycines (Mahoney et al., 1997). Numerous proteins contain such poly-proline motifs and were described as profilin ligands. Many profilin interacting proteins clearly belong in the category of actin regulators like Ena/VASP proteins, formins, and WASP/WAVE. Other PLP-ligands link profilin to diverse cellular processes such as membrane trafficking, signaling, synaptic scaffolding, nuclear export, mRNA splicing, and transcription (see Supplementary Table 1). In addition, profilin also interacts with phospholipids, while it has the highest affinity to phosphoinositide-4,5-bisphosphate (PIP_2_) (Lassing and Lindberg, 1985, 1988). The major phosphoinositide-binding pocket with its conserved basic residues overlaps with the actin binding site (Sohn et al., 1995; Behnen et al., 2009). Thus, PIP_2_-bound profilin is unable to interact with actin monomers (Lassing and Lindberg, 1988). Biochemical analyses also show the presence of an additional PIP_2_ binding site at the profilin C-terminus (Lambrechts et al., 2002; Skare and Karlsson, 2002). The competition of PIP_2_ with other profilin ligands serves as a regulative mechanism of profilin activity downstream of phospholipid-based signal transduction (see for review Davey and Moens, 2020).

## Profilin as Promoter and Director of Actin Dynamics

**FIGURE 2:**
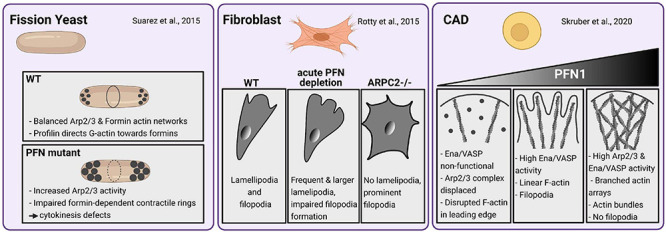
Profilin-dependent mechanisms in coordinating different actin networks vary in a cell type-specific manner. *Fission yeast (left panel)*: Profilin balances ARP2/3 and Formin-dependent actin networks by directing G-Actin toward formins enabling normal contractile ring development and, thus, normal cytokinesis. Under semi-permissive temperatures, PFN mutants show large actin patches by increased ARP2/3 activity and impaired contractile ring formation and cytokinesis defects according to formin dysfunctions (Suarez et al., 2015). Mouse Fibroblasts (c*enter panel)*: Wildtype fibroblasts form normal lamellipodia and filopodia. An acute depletion of PFN leads to increased F-Actin levels and formation of prominent ARP2/3-rich lamellipodia. Filopodia formation is impaired in PFN1-depleted fibroblasts. ARP2-deficient fibroblasts exhibit prominent filopodia but no lamellipodia. Actin dynamics in ArpC2-deficient cells rely on PFN1 and Ena/VASP-proteins (Rotty et al., 2015). *CAD cells (right panel)*: PFN deficiency disrupts the actin networks in leading edges of CAD cells by rendering Mena/VASP proteins inactive and displacing the Arp2/3 complex. Rescue experiments with low to medium levels of PFN1 mediate exclusively linear actin filaments and filopodia formation by high Mena/VASP-activity. High levels of profilin in these settings evoke both high Arp2/3 and Mena/VASP activity. Thus, dense branched actin arrays are formed along with linear actin bundles. The latter resembled filopodia precursors while actual filopodia were not formed (Skruber et al., 2020).

The roles of profilin in actin dynamics have been intensively investigated *in vitro* and in cells, mostly by studying either yeast profilin or mammalian profilin 1 (PFN1): in line with its first description as a protein that can keep actin in a “pro-filamentous state,” profilin was initially considered to maintain the pool of free actin monomers (Carlsson et al., 1977). However, numerous studies identified profilin instead as an actin polymerization-driving force by the following mechanisms: (1) profilin accelerates the nucleotide exchange of G-actin (Perelroizen et al., 1996; Selden et al., 1999), (2) ATP-G-actin-bound profilin can transiently bind to growing barbed ends of actin filaments (Jégou et al., 2011; Courtemanche and Pollard, 2013; Pernier et al., 2016) and (3) profilin delivers G-actin to actin-nucleating and/or polymerizing proteins through the interaction with poly-proline motifs (Ferron et al., 2007; Paul et al., 2008; Funk et al., 2019). Prominent classes of such actin regulators are formins and Ena/VASP proteins, which act as processive actin polymerases by continuously associating with profilin-actin complexes, thereby creating linear actin filaments (Romero et al., 2004; Breitsprecher et al., 2008; Hansen and Mullins, 2010; Winkelman et al., 2014; Brühmann et al., 2017). On the other hand, profilin also interacts with Arp2/3 complex-specific nucleation promoting factors (NPFs) like (N-)WASP and WAVE proteins (Miki et al., 1998; Suetsugu et al., 1998). These proteins activate the Arp2/3 complex, which then binds already existing filaments and initiates there the growth of actin branches (see for review Pollard and Cooper, 2009). In view of this promiscuous binding nature, one could interpret profilins as housekeeping proteins that equally provide polymerization-competent actin monomers to the different actin networks. However, recent studies demonstrate that profilin directs as master switch the competition of branched and linear actin networks for free actin monomers. In yeast, high profilin concentrations blocked Arp2/3-dependent actin networks, while formin-dependent actin polymerization is greatly enhanced. Profilin yeast mutants show increased Arp2/3-dependent actin patches and cytokinesis defects through formin dysfunctions (Figure 2; Suarez et al., 2015). In line with these findings are results obtained in fibroblasts, where acute profilin depletion in wildtype cells cause increased F-actin levels and formation of Arp2/3-rich lamellipodia (Rotty et al., 2015). In contrast, actin dynamics of ArpC2-deficient cells mostly rely on profilin and Ena/VASP proteins (Figure 2). These studies indicate an obvious supportive role of profilin for formin and Ena/VASP-based over Arp2/3-dependent actin networks. From a mechanistic point of view, profilin can inhibit branched actin networks indirectly by re-routing G-actin away from Arp2/3-specific NPFs toward formins and Ena/VASP. On the other hand, a direct inhibition of actin branching is conceivable, as profilin can principally interact with Arp2 and, thus, sterically hinder the Arp2/3 complex itself (Mullins et al., 1998) and/or associated NPFs like WASP (Suarez et al., 2015). However, it is important to note that other studies also showed stimulatory effects of profilin on N-WASP and WAVE1 as well as Arp2/3-dependent actin networks (Bieling et al., 2018; Skruber et al., 2020). Profilin 1-deficient CAD cells, which are of neural origin, show Arp2/3 network-deprived leading edges, while Ena/VASP proteins are non-functional at the same time. Rescue experiments with tightly titrated profilin levels reveal discrete stages of either competition or collaboration between the different actin networks: Low profilin levels in CAD cells favor exclusively linear filaments and filopodia formation at leading edges through Ena/VASP proteins. In contrast, high profilin levels evoke the Arp2/3-dependent branched actin arrays in conjunction with actin bundles resembling filopodia-precursor structures. However, no filopodia are generated in high profilin settings (Figure 2; Skruber et al., 2020). Therefore, the regulative function of profilin on different actin networks in CAD neuroblastoma cells is dosage-dependent. In fibroblasts, Rotty et al. show a clear preference of profilin in driving the formin and Ena/VASP dependent actin machinery. The discrepancies between both studies can be explained by differences in the used substrates and cell types: Primary fibroblasts on fibronectin show prominent stress fibers and several lamellipodia at the leading edge (Rotty et al., 2015). CAD cells on laminin form one continuous and large lamellipodium, where F-actin appears veil-like or in linear arrays of short actin bundles. In contrast to fibroblasts, CAD cells do not form stress fibers (Lee et al., 2013; Skruber et al., 2020). A major source of variations may be the used extracellular matrix proteins: cultivating the same cell type on either fibronectin or laminin can already cause profound differences in its cellular morphology and behavior (Junker and Heine, 1987; Siddiqui et al., 2021). Moreover, several studies demonstrate that neurons can use entirely different modes of migration than fibroblasts (Strasser et al., 2004; Yang et al., 2012; San Miguel-Ruiz and Letourneau, 2014; Santos et al., 2020): For instance, neuronal axons move in an amoeboid manner independent of adhesions and actomyosin mediated forces, while fibroblasts use contractility-based mesenchymal migration when cultivated in 3D matrices (Santos et al., 2020). Thus, the profilin-dependent mechanisms to control actin network architectures could vary between fibroblasts and neuron-derived CAD cells as well. In summary, profilin is a major driving force of actin polymerization and director of the different actin networks. However, the precise mechanisms in how profilin coordinates the different actin networks, appear to vary in a context- and cell type-dependent manner.

## Profilin Isoforms in Higher Animals

**FIGURE 3:**
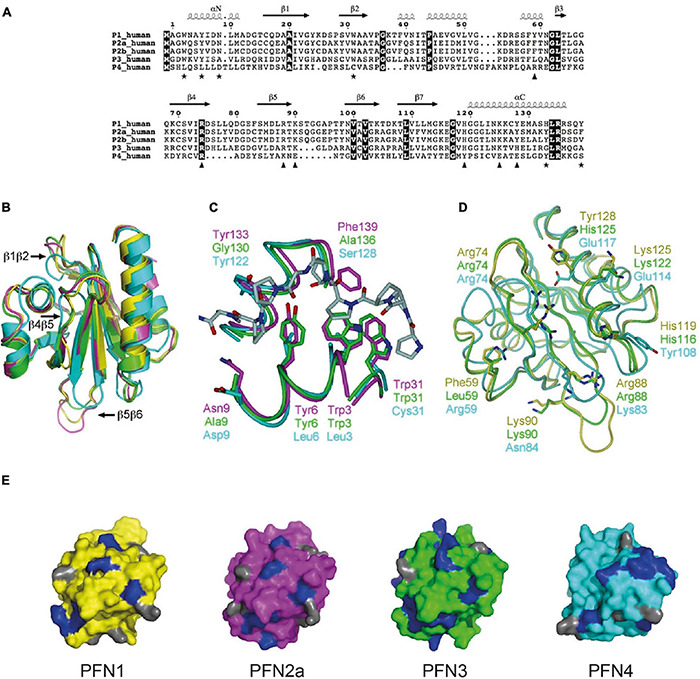
Structural features reflect the different biochemical properties of the mammalian profilin isoforms. **(A)** Sequence alignment between human profilins. Fully conserved residues are on dark background. Key residues of PLP binding are indicated by asterisks, those involved in binding actin with triangles. Secondary structures are derived from bovine PFN1 crystal structure. For compatibility with most publications, the first methionine is not considered in sequence numbering. **(B)** Superposition of bovine PFN1 (yellow), mouse PFN2a (magenta), human PFN3 (green), and human PFN4 (cyan). PFN1 structure is from the profilactin complex (PDB core 1HLU) and PFN2a from the complex with a VASP peptide (PDB code 2V8C). Loops variable in length in PFN3 and/or PFN4 are marked. **(C)** Comparison of the PLP-binding sites of PFN2a, PFN3, and PFN4. Shown is also the PFN2a-VASP complex, coloring as in 3B. Only half of the binding site is conserved in PFN3, no conservation is seen in PFN4. Key residues for peptide binding are indicated. **(D)** Actin-binding sites of PFN1, PFN3, and PFN4, coloring as in 3B. For clarity, actin is not shown; view is from the direction of actin onto the actin-binding surface on profilin, side chains of key profilin residues are shown. **(E)** Comparison of PtdIns(4,5)P2 binding surfaces of PFN1, 2a, 3, and 4 (left to right, respectively). Profilins are colored as in 3A, and all arginine residues, crucial for PtdIns(4,5)P2 binding, are highlighted in blue; lysine residues are shown in gray. This figure is used in this review with permission from Behnen et al. (2009).

In the previous chapters, we referred to “typical profilin properties” of which particularly the ability to bind actin and poly-proline motifs is widely used to classify proteins with corresponding structure as profilins. When we reviewed the regulation of actin dynamics by “profiling,” we discussed studies using either yeast profilin or mammalian profilin 1. In the following we shine some light on profilins in higher animals: So far, 4 profilin genes have been identified in vertebrates, where the alternative splicing of *pfn2* transcript produces two isoforms, PFN2a and PFN2b. PFN1 is ubiquitously expressed and essential for normal development, as early embryos die during the first cell divisions in PFN1-deficient settings or subsequent to injecting anti-PFN1 antibodies into zygotes (Witke et al., 2001; Rawe et al., 2006). In the CNS, PFN1 constitutes only 25% of total profilin protein levels. The majority is represented by the co-expressed PFN2a, which is, according to its expression pattern in mice, categorized as the CNS-specific profilin isoform (Witke et al., 1998). However, PFN2a is more broadly expressed in other organisms. For instance, PFN2a is the ubiquitous isoform in chicken (Murk et al., 2009). In addition, PFN2a is also present in non-neuronal cells and tissues of humans (Mouneimne et al., 2012). The other PFN2 splice form PFN2b is only expressed in kidney (Di Nardo et al., 2000). PFN3 and PFN4 are testis-specific isoforms, while PFN4 shows the lowest level of homology to the other isoforms (Figures 1, 3; Hu et al., 2001; Obermann et al., 2005).

Structural comparisons show that generally all isoforms possess the typical profilin folding. PFN4, however, is ten amino acids shorter than PFN1 and PFN2a, which reduces the lengths of three interspacing loops (β1β2, β4β5, and β5β6, Figures 3A,B). PFN4 interacts neither with G-actin nor poly-proline stretches, as it lacks all the necessary key residues to bind these typical profilin ligands (Figures 3A–C). Another difference is a shifted phospholipid binding site in PFN4, which enables it to bind PtdIns(4)P and phosphatidic acid *in vitro* (Figure 3E; Behnen et al., 2009). Analogous to PFN4, PFN2b does not have a significant affinity to G-actin and poly-proline motifs either (Di Nardo et al., 2000). Although the cellular functions of these profilin isoforms are currently unknown, it is conceivable that they fulfill different tasks than controlling actin dynamics. The other profilin isoforms vary more subtly in the typical profilin properties: The testis-specific PFN3 isoform interacts both with G-actin and poly-proline motifs but with significantly lower affinity than “the” PFN1. This different binding behavior is structurally reflected by conservative amino acid sequence differences in the actin binding sites of PFN1 and PFN3 (Figure 3D). In addition, the two key residues His133 and Phe139, which mediate high-affinity binding of PFN1 to poly-proline, are not present in PFN3 (Figures 3A,C). The putative phospholipid binding site of PFN3 is structurally comparable to PFN1 and PFN2a (Figure 3E; Behnen et al., 2009).

PFN2a, the predominant splice form of *pfn2*, has actin-binding properties comparable to PFN1 (Lambrechts et al., 1995, 2000), while it binds *in vitro* with higher affinity to synthetic poly-proline peptides (Lambrechts et al., 1995). These experimental findings are supported by structural data showing all residues central to actin binding are conserved between PFN1 and PFN2a (Figures 3A,D). The poly-proline binding domain of PFN2a is highly similar to PFN1, while the aromatic residues Tyr29 and Tyr133 in PFN2a further enhance its affinity to poly-proline (Figures 3A,C; Nodelman et al., 1999; Haikarainen et al., 2009; Walter et al., 2020). The phospholipid binding sites are well conserved between PFN1 and PFN2a (Figure 3E). However, the greater binding strength of PFN2a to poly-prolines prevents, in contrast to PFN1, a competitive binding between synthetic poly-proline peptides and PIP_2_ (Lambrechts et al., 1997). Both similarities and differences between PFN1 and PFN2a *in vitro* raise the question as to whether the isoforms are functionally exchangeable or fulfill different tasks in cellular settings.

## Profilin 1 vs. Profilin 2a – From Proteins, Mice and Chicken

To discuss the functional diversity of PFN1 and PFN2a, we here elaborate their binding properties to different ligands as well as the phenotypes, caused by manipulating PFN1 and/or PFN2a in cells and animals. For the latter, we mainly focus on neuronal cells, where both isoforms are co-expressed.

PFN1 and PFN2a share a large number of mutual ligands but also bind some ligands exclusively (Supplementary Table 1). For instance, affinity chromatography based screens identified tubulin among the exclusively PFN1-bound proteins (Witke et al., 1998). This finding was confirmed and extended by recent studies showing a tubulin binding site in PFN1 close to its actin binding domain. The proximity of both binding sites prevents PFN1 to interact with tubulin monomers in *in vitro* settings, when G-actin is present (Henty-Ridilla et al., 2017). However, PFN1 associates in different cell lines with tubulin, cytoplasmic microtubules, mitotic spindles and centromers (Grenklo et al., 2004; Nejedla et al., 2016; Henty-Ridilla et al., 2017; Nejedlá et al., 2021.) Moreover, the manipulation of cellular PFN1 levels also affects microtubule dynamics: Depletion of PFN1 evokes increased microtubule growth in B16f-melanoma cells (Nejedla et al., 2016). In neuronal cells, PFN1 mediates the opposite effect: The velocity of growing microtubules is three-fold increased in PFN1-overexpressing N2A neuroblastoma cells (Henty-Ridilla et al., 2017). Increased PFN1 levels promote axon growth and regeneration of neurons in cell culture and *in vivo* by accelerating microtubule growth, while depletion of PFN1 curbs these processes (Pinto-Costa et al., 2020). These findings are in line with *in vitro* assays showing the direct enhancement of microtubule growth by PFN1 (Henty-Ridilla et al., 2017). In addition, PFN1 coordinates microtubules with actin filaments in axons via formins (Pinto-Costa et al., 2020), which serve as a link between both filament systems (see for review Breitsprecher and Goode, 2013). In summary, PFN1 specifically balances microtubule dynamics in yet to be determined cellular settings and via so far unknown molecular mechanisms.

**FIGURE 4:**
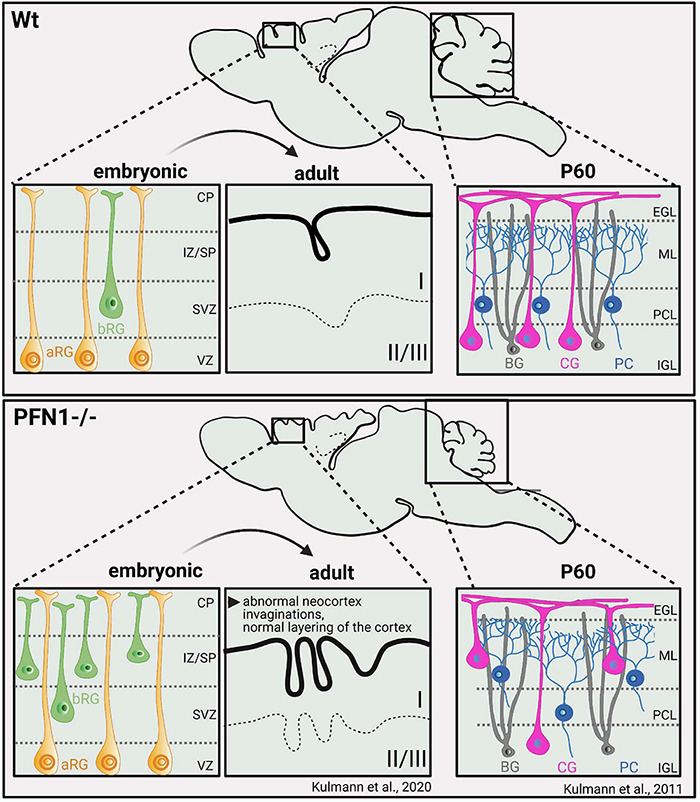
PFN1 loss perturbs brain development and causes malformations of cortex and cerebellum through altered cellular compositions. *Pfn1*^–/–^ mice exhibit smaller brains, with size reductions, particularly of the cortex (–25%) and cerebellum (–50%). Defects in cortical development are caused prenatally by a distinct subset of neural progenitor cells, known as basal radial glia. PFN1 loss affects the orientation of basal radial glia cell divisions during development, causing transiently ectopic neurogenesis. Adult mice show abnormal invaginations of the neocortex while cortical layering is principally preserved (Kullmann et al., 2020). Cerebellar hypoplasia develops postnatally by migration defects of cerebellar granule cells and Purkinje cells (Kullmann et al., 2011, 2012, 2015). Impaired adhesion and migration of cerebellar granule cells along the processes of Bergmann glia are causative for this phenotype. bRG, basal radial glia; aRG, apical radial Glia; CP, cortical plate; IZ/SP, intermediate zone/subplate; SVZ, subventiruclar zone; VZ, ventricular zone; BG- Bergmann Glia; CG- cerebellar granule cells; PC- purkinje cells; EGL, external granular layer; ML, Molecular layer; PCL, Purkinje cell layer; ICL, Internal granular layer; P60, postnatal day 60.

Loss of function studies show PFN1 to control distinct aspects in CNS physiology, which apparently are not compensated by the co-expressed PFN2a: The CNS-specific deletion of the *pfn1* gene causes specific anatomical changes in the mouse brain. PFN1-deficient mice exhibit smaller brains, where in particular the cortex with 25% and the cerebellum with up to 50% of size reduction are affected (Figure 4). The hypoplasia of the cerebellum develops postnatally, when the neuron subtypes of cerebellar granular neurons (CGNs) and Purkinje cells move toward their final positions. Firstly, CGNs and Purkinje cells migrate tangentially in the external granular layer. Then they switch to radial migration and move along the processes of Bergmann Glia to cross the molecular layer and reach their destinations, the Purkinje or internal granule layer inside the cerebellum (Chédotal, 2010). *Pfn1*-/- CGNs, however, fail to adhere and migrate along Bergmann glia fibers, which cause their ectopic accumulation in the intermediate molecular layer (Kullmann et al., 2011, 2015). Also, the co-migrating Purkinje cells show analogous migration defects. However, this phenotype turned out not to be cell autonomous, as the Purkinje cell-specific *pfn1* deletion does not reproduce the migration defect observed in mice with the CNS-wide ablation of *pfn1* (Kullmann et al., 2012). Thus, defective migration of PFN1-deprived cerebellar granule cells is somehow translated to migrating Purkinje cells. PFN1 deletion in the rodent CNS evokes impaired motor coordination, which is typical for disturbed cerebellum functions (Kullmann et al., 2012).

Defects in the cortex development of *pfn1-/-* mice occur prenatally (Figure 4; Kullmann et al., 2020). This finding is in line with a wealth of developmental studies showing that the cerebral cortex with its typical six horizontal layers and specialized neuron subtypes forms between embryonic day 10 and birth. The different neuronal cells in the cortex originate from radial glia cells in the ventricular and subventricular zone (Götz et al., 2002; Noctor et al., 2004). From there, radial glia cells and already differentiated neurons migrate and create the cortical layers in a stepwise manner (Gilmore and Herrup, 1997; López-Bendito and Molnár, 2003). In contrast to the developing cerebellum, the migration of radial glia cells and thus, cortical layering in PFN1 deficient brains is unaffected. *Pfn1-/-* brains display smaller and abnormal invagination-bearing cerebral cortices, a phenotype that can be attributed to cell division defects in basal radial glia cells, a rare subset of neural progenitor cells. Basal radial glia cells are highly neurogenic and can be found in the developing cortex of gyrencephalic species, where they are associated with the development of the cortical folds, known as gyri and sulci (Penisson et al., 2019).

**FIGURE 5:**
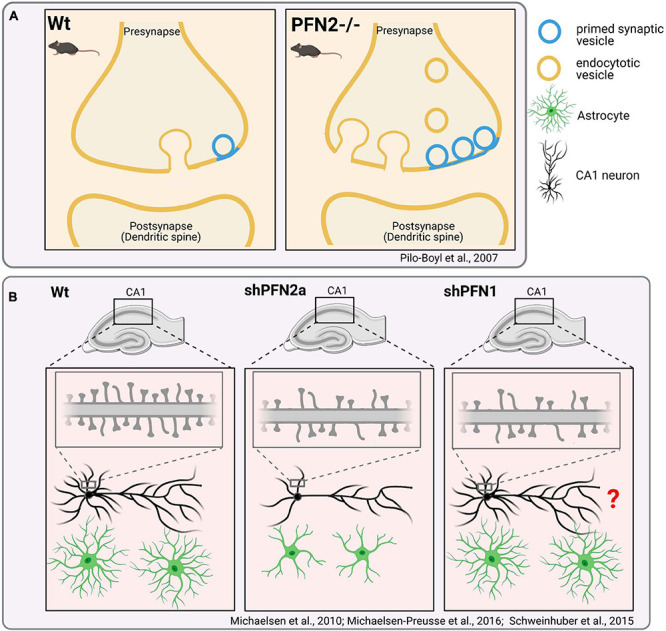
Distinct effects of PFN1- or PFN2- “loss-of-function” in neuronal cells. **(A)** Genetic ablation of Profilin2 and impact on synapses: Presynapses in *pfn2*-deficient mice show an increased number of primed synaptic vesicles. Increased exocytosis of synaptic vesicles cause hyperactivity and changed complex behaviors in *pfn2*-/- mice, such as increased novelty-seeking (Pilo Boyl et al., 2007). Further studies also indicate increased endocytosis in *pfn2*-/-mice (Gareus et al., 2006; Luscieti et al., 2017). *Pfn2* ^–/–^ animals display no gross changes in brain anatomy. **(B)** shRNA-mediated acute depletion of PFN2a in organotypic slice cultures reduces dendritic complexity and spine densities of hippocampal CA1 neurons (Michaelsen et al., 2010). shPFN1 treatment of neurons reduces the overall densities of mature dendritic spines (Michaelsen-Preusse et al., 2016). It was not described, if PFN1 knockdown also affects the dendritic arborization of CA1 neurons (labeled with “?”). Rescue experiments indicate that PFN1 is unable to compensate PFN2a-specific defects in dendritic complexity. Depletion of PFN2a but not PFN1 affects the overall volume of astrocytes in organotypic slice cultures (Schweinhuber et al., 2015).

In *pfn1*-/- brains, alterations in basal radial glia cell division result in locally increased and ectopic neurogenesis at embryonic day 14.5. Later in development, the atypical invaginations form in the cortex periphery after the ectopic basal radial glia cell-derived neuronal cells reached their final positions and differentiate in cortical layer I (Kullmann et al., 2020). In contrast to the distinct CNS deformities in this *pfn1* knockout mouse model, excitatory synapses were structurally and functionally intact (Görlich et al., 2012). However, it is noteworthy that the acute depletion of PFN1 in cultured hippocampal neurons and organotypic slices by RNAi affects dendritic spines, which harbor the majority of excitatory postsynapses (see for review Borovac et al., 2018). The PFN1 knockdown reduces the overall densities as well as the content of mature subtypes of dendritic spines (Figure 5B; Michaelsen-Preusse et al., 2016). Thus, PFN1 controls distinct aspects of brain development, while further research is required to clarify its role in synapses.

In contrast to PFN1, PFN2a binds membrane- and synaptic vesicle-associated proteins like synapsins and dynamin I in ligand screenings (Witke et al., 1998). Cell biological and *in vivo* studies with *pfn2*-deficient mice reveal accordingly functions of PFN2a in synaptic membrane trafficking: PFN2a is able to inhibit endocytosis through its interaction with dynamin I (Gareus et al., 2006). Moreover, PFN2a-deficient mice show increased synaptic exocytosis upon depolarization, which correlates with altered synaptic F-actin levels and larger number of primed synaptic vesicles in *pfn2-/-* synapses when compared to wildtype synapses (Figure 5A; Pilo Boyl et al., 2007). Interestingly, *pfn2-/-* mice do not show gross changes in brain anatomy. Instead they exhibit behavioral abnormalities such as hyperactivity and increased novel object seeking. This phenotype correlates with electrophysiological analyses showing increased synaptic excitability of glutamatergic neurons in the striatum of *pfn*2-/- mice. The findings support the hypothesis that PFN2a tunes the presynaptic neurotransmitter release of neurons via actin dynamics. In turn, PFN2 loss creates the over-activation of neural circuits like the cortico−striatal glutamatergic pathway, which relays its increased synaptic input to basal ganglia and thereby causes hyperactivity and altered complex behaviors. Thus, studies in *pfn*2-/- mice reveal a prominent role of PFN2a in presynapses. However, these findings do not rule out postsynaptic functions of PFN2a. NMDA receptor stimulation of cultured hippocampal neurons expressing PFN2a-GFP evokes the translocation of PFN2a into dendritic spines, where actin-dependent changes in spine shape are subsequently blocked (Ackermann and Matus, 2003). Fear conditioning of rats induces the translocation of endogenous profilin into dendritic spines of the amygdala (Lamprecht et al., 2006). Also, localization studies with a PFN2-specific antiserum confirm the presence of PFN2a in dendritic spines of cultured neurons depending on synaptic activity and Rho signaling (Schubert et al., 2006). Finally, acute PFN2a depletion in organotypic slice cultures reduces the dendritic complexity and spine densities of hippocampal CA1 neurons (Figure 5B). The concomitant expression of exogenous PFN1 rescues defects in dendritic spine density but not in dendrite arborization of PFN2a-depleted CA1 neurons (Michaelsen et al., 2010). These results demonstrate functions of PFN2a in postsynaptic and dendritic compartments, which partially can be compensated for by upregulated PFN1.

In addition, PFN1 and PFN2a are also involved in extra-synaptic functions in the CNS. Both isoforms are expressed in astrocytes, the most prevalent and highly arborized glia cells in the CNS, which actively support and protect neurons (see for review Schiweck et al., 2018). Depletion of PFN2a, but not PFN1, reduces the total volume that astrocytes occupy in organotypic brain slices (Figure 5B; Schweinhuber et al., 2015). PFN1 knockdown in cultured astrocytes instead affects the number and motility of filopodia-like protrusions, which resemble dynamic perisynaptic processes of astrocytes *in vivo* (Molotkov et al., 2013). Another recent study shows that PFN2a is involved in iron homeostasis. The *pfn2* transcripts contain an iron response element in their 3′-UTR, which positively regulates *pfn2* mRNA stability upon binding by iron response proteins. PFN2 deficiency leads to excessive iron accumulation specifically in the brain, possibly through increased transferrin uptake by deregulated endocytosis (Luscieti et al., 2017). Thus, PFN1 and PFN2a possess mutual as well as isoform-specific functions in the CNS, which regulate multiple processes including membrane trafficking and shaping of the plastic morphology of neuronal cells.

However, the question remains whether PFN2a can also fulfill tasks of PFN1 in non-neuronal cells. To our knowledge, no direct gene replacement of endogenous PFN1 with PFN2a in such settings has been published. However, chicken ubiquitously express PFN2a (chPFN2a), which, surprisingly, differs from murine PFN2a by a single amino acid substitution at position 38 (Murk et al., 2009). In chicken fibroblasts, PFN2a is responsible to control actin dynamics in general. Chicken PFN2a is co-expressed with a PFN1-like isoform (chPFN1). Sequence alignments with mouse PFN1 showed, however, that chPFN1 differs largely in residues, which are otherwise conserved between mammalian PFN1 isoforms and are crucial for actin binding (Schlüter et al., 1998). Functional assays with chicken fibroblasts after single and double knockdown of chPFN1 and/or chPFN2a demonstrate that chPFN1 loss has no significant impact on actin-dependent processes like cell adhesion, spreading, and migration (Murk et al., 2009). Thus, PFN2a can principally exert functions in non-neuronal cells so far ascribed to PFN1. Still, it is currently unknown if PFN2a has additional functions in chicken fibroblasts, such as the regulation of membrane trafficking events.

## Profilin 1 and Profilin 2a in Neurological Pathologies

Growing numbers of publications, studying patients and/or using cell or animal disease models, link profilins to various diseases: in cancers, PFN1 as well as PFN2 are reported to either act as tumor suppressors or possess oncogenic potential, depending on the studied cancer cell type (for review Pimm et al., 2020). In addition, some studies link PFN1 to a degenerative bone pathology known as Paget Disease (Merlotti et al., 2020; Scotto di Carlo et al., 2020), while others implicate this profilin isoform in vascular inflammation and atherosclerosis (see for review Pae and Romeo, 2014). Particularly, studies on neurological diseases emphasize both the biomedical relevance and the functional diversity of PFN1 and PFN2a isoforms. Therefore, in the following we will focus on the roles of PFN1 and/or PFN2a in a subset of prominent neurological pathologies comprising Amyotrophic lateral sclerosis (ALS), Fragile X syndrome (FXS), Huntington’s disease (HD), and spinal muscular atrophy (SMA).

## Amyotrophic Lateral Sclerosis

Amyotrophic lateral sclerosis (ALS) is a major neurodegenerative disease, which is caused by mutated *Pfn1* alleles (Wu et al., 2012; Smith et al., 2015). This disease is characterized by late-onset progressive degeneration of motor neurons in the brain and spinal cord, leading to impaired motor coordination, paralysis and, ultimately, to death by respiratory failure usually within 3 or 5 years from diagnosis. ALS cases are divided into two major groups - sporadic (sALS) and familial (fALS), while familial cases of ALS represent roughly 10% of total ALS cases (see for review (Hulisz, 2018). Exome sequencing studies reveal 8 mutations in the PFN1 gene in both familial and sporadic ALS cases (C71G, G118V, M114T, E117G, T109M, R136W, A20T, Q139L) (Figure 6.1; Wu et al., 2012; Chen et al., 2013; Ingre et al., 2013; Smith et al., 2015; Alkam et al., 2017). The PFN1-associated ALS pathology is reproducible in several mouse and rat models (Yang et al., 2016; Fil et al., 2017; Barham et al., 2018; Brettle et al., 2019; Yuan et al., 2020). The expression of ALS-PFN1 mutants evokes cytoskeletal and morphological defects in primary neurons, such as abnormally low ratios of F-/G-actin, shorter dendrites and integrity-impaired axons, which undergo Wallerian degeneration over time (Figure 6.2; Wu et al., 2012; Yang et al., 2016; Fil et al., 2017). Different scenarios are currently discussed in the field on how PFN1 mutations could evoke ALS: in view of PFN1’s central role in actin dynamics, one hypothesis is the perturbation of G-actin binding of PFN1 by ALS-associated mutations. The initially discovered mutations C71G, G118V, M114T, and E117G are in close proximity to the PFN1 G-actin binding site. Co-immunoprecipitation assays first indicated an impaired G-actin binding of the PFN1 C71G, PFN1 G118V, and PFN1 M114T mutants (Wu et al., 2012). However, subsequent analyses using recombinant proteins in pyrene-based actin polymerization assays demonstrate that none of those four PFN1 mutants is significantly affected in its binding to G-actin (Boopathy et al., 2015). In addition, the later discovered PFN1 T109M mutant also exhibits a normal affinity to G-actin (Freischmidt et al., 2015). Thus, the direct interaction of ALS-PFN1 mutants with G-actin is unlikely to cause the ALS pathology. Several analyses show that ALS-associated PFN1 mutants are prone to aggregate like the other ALS-causing proteins, such as SOD1, TDP43, and FUS (see for review Saberi et al., 2015). Some ALS-linked PFN1 mutations (C71G, M114T, G118V) form insoluble and ubiquitinated protein aggregates and inclusions in primary motor neurons and N2A cells, while control wildtype PFN1 is diffusely distributed across the cytoplasm (Figure 6.3; Wu et al., 2012). Also, the more recently identified PFN1 A20T variant causes the formation of insoluble aggregates in HEK293T cells and ALS patients-derived fibroblasts (Smith et al., 2015). Comprehensive biochemical analyses demonstrate the mutation-induced destabilization of protein folding in PFN1 C71G, PFN1 M114T, and PFN1 G118V variants (Boopathy et al., 2015; Del Poggetto et al., 2015a, b, 2016, 2018). These studies support the hypothesis that ALS-PFN1 mutants acquire different, partially or alternatively folded conformations, which promote the formation of PFN1 inclusions in the cytosol. X-ray crystallography reveals corresponding structural perturbations in aggregation-prone ALS-PFN1 mutants: the M114T mutation turns a surface pocket of wildtype PFN1 into a cleft, which extends into the protein core. Accordingly, this structural change exposes in wildtype PFN1 buried hydrophobic residues to solvent and, thereby, destabilizes the overall protein stability. Computer modeling suggests also a cavity deep within the PFN1 C71G mutant (Boopathy et al., 2015). Such internal voids are structurally more disruptive than peripheral, solvent-exposed cavities, which explains the particularly high instability and aggregation propensity of PFN1 C71G among the biochemically characterized ALS-PFN1 mutants (Eriksson et al., 1992b, a; Del Poggetto et al., 2015a; Xue et al., 2019). In line with the structural and biochemical findings is the particularly high susceptibility of PFN1 C71G to proteasomal degradation in cells (Schmidt et al., 2021). Interestingly, the PFN1 E117G and PFN1 Q139L mutants are less prone to aggregate (Smith et al., 2015). Structural comparisons also reveal a folding of PFN1 E117G highly similar to wildtype PFN1 (Boopathy et al., 2015). Both PFN1 E117G and PFN1 Q139L have in common to cause only mild phenotypes in cell models (Wu et al., 2012; Figley et al., 2014). PFN1 E117G is also under debate of rather being a benign polymorphism or a potential ALS risk factor, as it is also present in control patients (Fratta et al., 2014; Smith et al., 2015). Therefore, the mutation-dependent destabilization of PFN1 toward aggregation-prone conformations is required to drive the full-scale ALS pathology.

**FIGURE 6:**
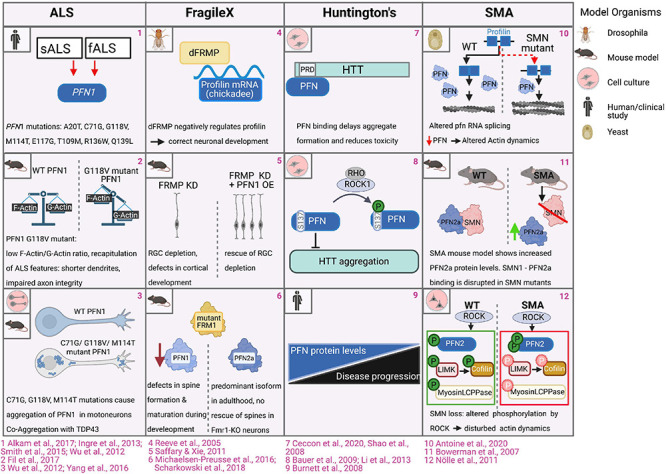
Profilins in CNS pathologies. **1.** Exome sequencing studies revealed 8 mutations in the *Pfn1* gene in familial and sporadic ALS patients (Ingre et al., 2013; Wu et al., 2012; Chen et al., 2013; Smith et al., 2015; Alkam et al., 2017). **2.** Motoneurons expressing ALS mutant PFN1 G188V exhibit abnormally low ratios of F-/G-actin. Motor neurons of ALS-PFN1-mutant expressing mouse model exhibited cytoskeletal disruptions and impaired axon integrity followed by Wallerian degeneration (Yang et al., 2016). **3.** In cell culture and mice, wildtype PFN1 is diffusely distributed across the cytoplasm of motor neurons and N2A cells, while PFN1 C71G, PFN1 M114T and PFN1 G118V PFN mutations form protein aggregates and inclusions. PFN1 co-aggregates with TDP43 (Wu et al., 2012; Yang et al., 2016). **4**. FRMP directly binds the mRNA of *Drosophila* profilin (encoded by *chickadee*), which is required for correct neuronal development in *Drosophila* (Reeve et al., 2005). **5**. FRMP knockdown in mice causes depletion of radial glia cells (RGC) and defects in cortical development, while overexpression of PFN1 in these settings restores the radial glial dependent developmental defects (Saffary and Xie, 2011). **6**. Levels of PFN1, but not PFN2a, are reduced in FRM1 mutant mice. Depletion of PFN1 causes defective dendritic spine formation and maturation during development analogous to defects in *Fmr1*-KO mice. Overexpressed PFN1, but not PFN2a, can rescue dendritic spine defects in *Fmr1*-KO neurons (Michaelsen et al., 2010; Scharkowski et al., 2018). **7.** Binding of PFN to the proline-rich domain of huntingtin reduces huntingtin aggregation and toxicity (Shao et al., 2008; Ceccon et al., 2020). **8.** Rho kinase-dependent phosphorylation of PFN1 at serine 137/8 enhances huntingtin aggregation. Pharmacological inhibition of ROCK inhibits huntingtin aggregation and toxicity (Bauer et al., 2009; Li et al., 2013). **9.** In humans, reduced profilin protein levels correlate with disease progression of Huntington disease in patients (Burnett et al., 2008). **10.** *smn*-deficient fission yeast displays splicing defects in the profilin gene, resulting in altered actin network homeostasis, perturbed cytokinesis and endocytosis (Antoine et al., 2020). **11.** Loss of SMN in SMA settings is associated with the upregulation of PFN2a protein levels (Bowerman et al., 2007). **12.** Levels of phosphorylated PFN2a are increased in SMA settings while ROCK-dependent phosphorylation of myosin light chain protein phosphatase (Myosin LCPPase) and the cofilin-regulating LIM-kinase, are reduced, causing disturbed actin dynamics (Nölle et al., 2011).

Moreover, ALS-PFN1 mutants frequently co-aggregate with TDP43, which on its own can cause ALS and Fronto-temporal dementia when aggregated (see for review Suk and Rousseaux, 2020). Co-aggregated mutated PFN1 and TDP43 possess prion-like properties and act as seeds that trigger the conversion of endogenous TDP43 into toxic conformational states when taken up by wildtype cells (Tanaka et al., 2016). In *Drosophila melanogaster*, ectopic PFN1 C71G and PFN1 M114T provoke increased neurodegeneration of retinal photoreceptor neurons via co-expressed TDP43 (Matsukawa et al., 2016). Thus, PFN1 aggregations exacerbate the course of ALS by increasing the toxicity of TDP43.

While the findings showing the toxic PFN1 aggregates are compelling, is it still unclear if also monomeric mutant PFN1 proteins contribute to the ALS pathology. In fact, one study reports accelerated motor neuron degeneration in PFN1 C71G expressing mice prior to the occurrence of PFN1 inclusions (Yang et al., 2016). One potential mechanism via monomeric PFN1 mutants could be the regulation of microtubule dynamics: unlike wildtype PFN1, four ALS PFN1 mutants (C71G, M114T, E117G, G118V) are unable to accelerate the growth of microtubules *in vitro* and in N2A cells (Henty-Ridilla et al., 2017). These findings correlate with the impaired axon integrity and subsequent Wallerian degeneration in mutant PFN1-expressing motor neurons (Yang et al., 2016; Fil et al., 2017). Another possibility could be changes in the activity of specific-PFN1 binding partners involved in actin dynamics: PFN1 M144T and PFN1 G118V mutants bind, with higher affinity than wildtype PFN1, the formin proteins DIAPH1 and 2 and FMNL1. Moreover, PFN1 M144T and PFN1 G118V significantly enhance the actin assembly rate of those formins. The augmenting function of both mutations relies on a heightened flexibility in the PFN1 α4 helix, which contacts the actin as well as the poly-proline binding site (Schmidt et al., 2021). In addition, disturbances in nuclear pore complexes and nuclear-cytoplasmic transport occur in ALS PFN1 mutants expressing neurons (Giampetruzzi et al., 2019). Whether this phenomenon is a primary effect of mutant PFN1 or caused indirectly, is currently unknown. In summary, ALS-associated PFN1 mutants possess toxic gain-of-function of which particularly the formation of amyloid-like protein aggregates with TDP43, but potentially, also additional properties of monomeric PFN1 mutants, cause the ALS pathology. Nevertheless, many open questions remain regarding the molecular pathomechanisms and the reason why specifically motor neurons degenerate through mutations in a ubiquitous actin binding protein.

## Fragile X Syndrome

Animal models show the association of profilin with the Fragile-X syndrome (FXS), one of the most prevalent neurodevelopmental disorders. Cardinal symptoms in the spectrum of FXS are considerable intellectual disabilities, hyperactivity and repetitive behaviors. Frequently, clinical signs of autism may occur in FXS patients, such as reduced social interactions, and impairments of speech. Causative for this X-chromosome-linked disease are mutations in the *FMR1* gene affecting the expression and/or function of the Fragile X mental retardation protein (FMRP). As a RNA-binding protein, FMRP binds and regulates the localization, stability and translation of multiple transcripts including those of key regulators controlling neuronal development, morphology, and synaptic plasticity (see for review Bagni and Zukin, 2019). The initial findings connecting FXS to profilin were obtained in *Drosophila*: FRMP directly binds the mRNA of *Drosophila* profilin (encoded by *chickadee*), while FMRP mutant flies expressed significantly increased levels of profilin (Figure 6.4; Reeve et al., 2005). The FMRP-dependent regulation of profilin expression occurs in flies at a late developmental stage, when sensory-input leads to a refinement of neural circuits through limiting of axonal growth and pruning of axonal branches (Tessier and Broadie, 2008). While findings in *Drosophila* indicate a negative role of FMRP in regulating profilin expression, studies in mice came to opposite conclusions: overexpression of PFN1 in radial glial cells largely rescues developmental defects during corticogenesis in FMRP-depleted mice (Figure 6.5; Saffary and Xie, 2011). In view of profilin isoform specificity, protein levels of PFN1, but not PFN2a, are reduced in FMR1-mutant mice. Depletion of PFN1 also leads to defective dendritic spine formation in hippocampal neurons, which correlate with corresponding structural alterations in FMRP-deficient mice (Figure 6.6; Michaelsen-Preusse et al., 2016; Scharkowski et al., 2018). While PFN1 protein expression is apparently affected in FMR1-mutant mice, it is not clear, whether these effects are caused by a direct regulation of mouse PFN1 transcripts by FMRP or are indirect: one study reports the direct interaction between FMRP and the PFN1 transcript (Michaelsen-Preusse et al., 2016), while large screens did not detect PFN1 transcript as FMRP target (Brown et al., 2001; Miyashiro et al., 2003; Darnell et al., 2011). Whereas the discussed studies demonstrate the implication of PFN1 in animal models, it is to our knowledge unknown, if PFN1 is also affected in FXS patients.

## Huntington’s Disease

Huntington’s disease (HD) is an autosomal dominant, progressive neurodegenerative disease caused by an expanded CAG repeat/polyglutamine (polyQ) tract beyond 35 repeats within the first exon of the huntingtin (*HTT*) gene. The physiological functions of the wildtype huntingtin protein are not yet fully understood, as it participates in various processes during development and adulthood, like cell survival, membrane trafficking, ciliogenesis, autophagy and transcription (see for review Saudou and Humbert, 2016). However, mutated huntingtin proteins with N-terminal elongated polyglutamine sites are prone to form toxic aggregates of oligomers and fibers. By these means, mutant huntingtin also impairs the correct function of co-expressed wildtype huntingtin protein to maintain survival and normal activity of neurons. The consequence of carrying toxic huntingtin mutations is comprehensive neurodegeneration particularly in the striatum, which affects neuronal circuits innervating basal ganglia and the cortex. Typical HD symptoms therefore comprise adult-onset progressive motor dysfunctions, personality changes with progressive emotional and psychiatric disturbances as well as cognitive decline (Zuccato et al., 2010). Huntingtin contains two prolin-rich domains (PRDs), which are binding sites for PFN1 and PFN2 (Figure 6.7; Shao et al., 2008). The interaction with profilins significantly reduces huntingtin aggregation and toxicity (Posey et al., 2018; Ceccon et al., 2020): profilins maintain huntingtin in soluble states and, thus, delay its clustering into first nucleation seeds. On one hand, binding of PFN1 to the PRD region of huntingtin abrogates its capability to form aggregation-prone tetramers. On the other hand, PFN1 also stabilizes huntingtin monomers and dimers, which don’t oligomerize further (Ceccon et al., 2020). However, profilins lose their attenuating effect on huntingtin toxicity, once larger aggregates are formed. The interaction with huntingtin is negatively regulated by the Rho kinase-dependent phosphorylation of PFN1 at serine 138 within its poly-proline binding area (Figure 6.8; Shao et al., 2008; Shao and Diamond, 2012). Pharmacological inhibition of ROCK accordingly inhibits huntingtin aggregation and toxicity in cell culture and animal models (Bauer et al., 2009; Li et al., 2013), which alters profilin activity along with other ROCK target proteins (Narayanan et al., 2016). These biochemical and cell biological findings are in line with a comparative study showing the correlation of reduced profilin levels with the progression of Huntington disease in patients (Figure 6.9; Burnett et al., 2008).

## Spinal Muscular Atrophy

The survival of motoneuron 1 protein (SMN1) is another ligand that links profilin to a major neurological disorder known as spinal muscular atrophy (SMA). SMN is probably best known for its roles in RNA splicing by assembling and guiding small nuclear riboproteins (snRNPs). However, they broadly participate in various processes, such as RNA metabolism, mRNA trafficking and localization, cell signaling, and also cytoskeletal dynamics (see for review Singh et al., 2017). Deletions or mutations in the human *SMN1* gene cause SMA, leading to progressive loss of lower motoneurons followed by muscle atrophy. SMA varies in its onset and progression, while severe forms develop prenatally and are frequently fatal for infants. Patients with less severe SMA vary in their symptoms ranging from cases, who experience muscle weakness to never being able to walk. The pathogenesis and severity of SMA relies on the type of mutations in the *SMN1* gene and the homologous *SMN2*. The *SMN2* isoform is rather unstable through the exclusion of exon7 and cannot fully compensate *SMN1*. However, increased SMN2 protein levels can render SMA to its milder courses (see for review Kolb and Kissel, 2015). SMN protein interacts with cytoskeletal proteins (Fallini et al., 2012), suggesting that cytoskeleton dynamics might be perturbed in SMA. In particular, SMN1 interact with both PFN1 and PFN2a, while it binds stronger to PFN2a (Giesemann et al., 1999). Pathogenic missense mutations or deletions of exon 5 or 7 in *SMN1* disrupt the interaction with PFN2a (Sharma et al., 2005). Mapping the SMN protein shows C-terminal poly-proline motifs being critical for the interaction with profilins. Moreover, endogenous PFN1 and PFN2a co-localize with SMN in neurite-like processes of PC12 cells and nuclei of motoneurons (Giesemann et al., 1999; Sharma et al., 2005). *In vitro*, SMN attenuates PFN2a’s ability to inhibit actin polymerization assays using pyrene-labeled G-actin (Sharma et al., 2005). These findings implicate a direct regulative role of SMN on profilin functions in actin dynamics. Moreover, other studies indicate a direct regulative role of SMN on profilin protein levels and function: Analyses of *SMN*-deficient fission yeast show splicing defects in the profilin gene. The SMN-dependent splice defect decreases profilin protein levels resulting in tilted actin network homeostasis, which translate into perturbed cytokinesis and endocytosis in fisson yeast (Figure 6.10; Antoine et al., 2020). However, it is noteworthy that fission yeast expresses only one profilin isoform, which may differ in its regulation by SMN from the mammalian isoforms. Accordingly, depletion of SMN in rat PC12 cells causes an increase in PFN2a protein (Figure 6.11; Bowerman et al., 2007). Furthermore, reduced SMN levels affect post-translational modifications of PFN2a: This profilin isoform is hyper-phosphorylated at multiple sites within its PLP-binding site, when SMN is depleted in cell lines and in spinal cords of a SMA mouse model (Nölle et al., 2011). These findings implicate that the lack of SMN-PFN2a complexes increases the accessibility of PFN2a to its upstream Rho kinase (ROCK). While levels of phosphorylated PFN2a are increased in SMA settings, the phosphorylation of other ROCK targets, such as the myosin light chain protein phosphatase (Myosin LCPPase) and the cofilin-regulating LIM-kinase, are reduced (Figure 6.12). Such changes in ROCK-dependent phosphorylation implicate misbalanced activities of PFN2a, myosins, and cofilin in SMN-deficient cells. The consequences are likely perturbed cytoskeletal dynamics in line with observed changes in F/G-actin ratio, formation of stress-related actin rods and impaired neurite outgrowth in SMN-depleted PC12-cells and SMA motoneurons (Nölle et al., 2011; Walter et al., 2021). Thus, erratic PFN2a levels and activity correlate with the pathogenesis of SMA but further investigations are necessary to clarify the molecular mechanism involved.

## Concluding Remarks

In this article, we provided an overview of general profilin features and reviewed the substantial progress made in studying the structural, biochemical, and functional properties of the different profilin isoforms in mammals. We focused on the functional diversity of PFN1 and PFN2a in the central nervous system under healthy and pathological conditions. However, our review also shows that we are still at the beginning of understanding the diverse roles that profilin and its isoforms play in cellular contexts. For many described profilin ligands we still do not know the impact that the interaction with profilins has on the corresponding cellular processes. Moreover, the functions of three out of five mammalian profilin isoforms are unknown, while discrepancies regarding the isoform-specific roles of PFN1 and PFN2a wait to be resolved. In view of profilin-associated diseases, more research is required to understand the involved molecular mechanisms behind the pathologies. Future research that tackles these open questions will immensely profit from newly developed methods and tools. The increasingly improving CRISPR/Cas-based methods will open new avenues to study profilins by loss of function, gene-tagging, and gain of function experiments in a great variety of cell types and organisms (see for review (Pickar-Oliver and Gersbach, 2019). Particularly tagging of endogenous PFN1 will be feasible by new profilin constructs internally fused to fluorescent proteins (Nejedla et al., 2017), as they circumvent the impaired ligand binding of traditional GFP-fused PFN1 variants (Geese et al., 2000; Wittenmayer et al., 2000). A better insight into the functions of profilin and its isoforms will help us to understand fundamental cell biological mechanisms in health and disease.

## Author Contributions

KM, MO, and JS wrote the manuscript. JS created the figures. All authors contributed to the article and approved the submitted version.

## Conflict of Interest

The authors declare that the research was conducted in the absence of any commercial or financial relationships that could be construed as a potential conflict of interest.

## Publisher’s Note

All claims expressed in this article are solely those of the authors and do not necessarily represent those of their affiliated organizations, or those of the publisher, the editors and the reviewers. Any product that may be evaluated in this article, or claim that may be made by its manufacturer, is not guaranteed or endorsed by the publisher.
