# Supplementary material for: Profilin Isoforms in Health and Disease – All the Same but Different
Source: Front Cell Dev Biol. 2021 Aug 12;9:681122. doi: 10.3389/fcell.2021.681122 (PMC8387879; doi:10.3389/fcell.2021.681122)
Supplement: Supplementary file 1 [file Table_1.DOCX]

Supplementary Material

# Supplementary Table S1: Ligands of PFN1 and PFN2a (without G-actin)

| **Profilin Ligand** | **Known Functions** | **interaction with**  **PFN1 and/or PFN2a** | **References** |
| --- | --- | --- | --- |
| vasodilator-stimulated phosphoprotein (VASP) | processive actin polymerase | PFN1, PFN2 | (Reinhard et al., 1995) |
| Ena, Mena | processive actin polymerase | PFN1, PFN2 | (Gertler et al., 1996; Ahern-Djamali et al., 1999) |
| EVL | processive actin polymerase | PFN2 > PFN1 | (Lambrechts et al., 2000; Mouneimne et al., 2012); |
| Formins | Processive Actin nucleation/polymerization | PFN1, PFN2a | see for review (Courtemanche, 2018) |
| N-WASP, WAVE1 | Nucleation promoting factors specific to Arp2/3 | PFN1, PFN2a | (Suetsugu et al., 1998, 1998; Bieling et al., 2018) |
| Cortactin | weak Arp2/3 complex NPF *in vitro*, stabilizes Arp2/3-actin arrays, actin dynamics, membrane trafficking | PFN1 | (Wang et al., 2014) |
| NCKAP1 (HEM2) | Component of WAVE complex, actin dynamics | PFN2a | (Witke et al., 1998) |
| Tubulin | Constituent of microtubules | PFN1 | (Witke et al., 1998) |
| Palladin | actin binding protein | PFN1 | (Boukhelifa et al., 2006) |
| Rap1 interacting adaptor molecule (RIAM) | links Rap1 GTPase to integrin activation, actin dynamics | PFN1 | (Lafuente et al., 2004) |
| Drebrin | F-actin binding & stabilizing protein | PFN1, PFN2a | (Mammoto et al., 1998) |
| CYFIP1 | Component of WAVE-Complex, FMRP and translation initiation factor 4E, Cytoskeletal dynamics & translation | PFN2a | (Witke et al., 1998) |
| NAA80 | Actin-specific N-terminal acetyltransferase | PFN2a >(?) PFN1 | (Rebowski et al., 2020; Ree et al., 2020) |
| Citron-N | Golgi organisation in neurons | PFN2a | (Camera et al., 2003) |
| synapsins Ia, Ib, Ia, IIb | organisation and regulation of synaptic vesicles | PFN2a | (Witke et al., 1998) |
| clathrin heavy chain | endocytosis | PFN1 | (Witke et al., 1998) |
| valosine containing protein (VCP) | Vesicle transport | PFN1 | (Witke et al., 1998) |
| Chloride intracellular channel 4 (CLIC4) | actin dynamics, membrane trafficking | PFN1 | (Argenzio et al., 2018) |
| Dynamin 1 | Scission of endocytic vesicles | PFN2a | (Gareus et al., 2006) |
| Rho dependent kinase (ROCK) | Kinase, promotes myosin based-contractility via myosin light chain phosphorylation, indirectly inactivates cofilin via phosphorylation of LIM kinase | PFN1, PFN2a | (Witke et al., 1998; Shao et al., 2008) |
| hPIV-2 V | Human parainfluenza virus type 2 protein, Rho signaling | PFN2 | (Ohta et al., 2019) |
| retinoic receptor alpha ( RARα) | nuclear receptor & transcription factor | PFN2a | (Andriamoratsiresy et al., 2018) |
| PTEN | Lipid & protein phosphatase, PI-3-Kinase antagonist | PFN1 | (Zaidi and Manna, 2016) |
| GluK2b | Ionotropic glutamate receptor, synaptic plasticity | PFN2a | (Mondin et al., 2010) |
| Gephyrin | Molybdenum cofactor synthesis, postsynaptic scaffold protein at inhibitory synapses | PFN1, PFN2a | (Giesemann et al., 2003) |
| NCAM | Neural cell adhesion, migration,neuritogenesis, synaptogenesis, synaptic plasticity | PFN2 | (Huang et al., 2020) |
| aczonin | scaffolding protein of presynaptic active zones | PFN1, PFN2 | (Wang et al., 1999) |
| Huntingtin | causative for Huntington's disease | PFn1, PFN2a | (Shao et al., 2008) |
| TDP43 | hnRNP protein, TDP43 accumulations cause ALS and Frontotemporal dementia | PFN1 | (Tanaka et al., 2016) |
| survial of motoneuron protein 1 (SMN1) | RNA splicing & metabolism, RNA localization, actin dynamics etc. Mutations cause spinal muscular atrophy (SMA) | PFN1, PFN2a | (Giesemann et al., 1999; Sharma et al., 2005) |
| Prrp | hnRNP, mRNA localization | PFN1 | (Zhao et al., 2001) |
| p80 Coilin | pre-mRNA splicing & maturation | PFN1 | (Skare et al., 2003) |
| p42POP/MyPOP | Myb-related transcription factor, tumor suppressor | PFN1, PFN2a | (Lederer et al., 2005; Wüstenhagen et al., 2018) |
| Exportin 6 | Nuclear export of G-actin-Profilin complexes | PFN1, PFN2a | (Stüven et al., 2003) |

**References**

Ahern-Djamali, S. M., Bachmann, C., Hua, P., Reddy, S. K., Kastenmeier, A. S., Walter, U., et al. (1999). Identification of profilin and src homology 3 domains as binding partners for Drosophila enabled. *Proc. Natl. Acad. Sci. U. S. A.* 96, 4977–4982. doi:10.1073/pnas.96.9.4977.

Andriamoratsiresy, D., Piskunov, A., Lutzing, R., and Rochette-Egly, C. (2018). PFN2a, a new partner of RARα in the cytoplasm. *Biochem. Biophys. Res. Commun.* 495, 846–853. doi:10.1016/j.bbrc.2017.11.096.

Argenzio, E., Klarenbeek, J., Kedziora, K. M., Nahidiazar, L., Isogai, T., Perrakis, A., et al. (2018). Profilin binding couples chloride intracellular channel protein CLIC4 to RhoA-mDia2 signaling and filopodium formation. *J. Biol. Chem.* 293, 19161–19176. doi:10.1074/jbc.RA118.002779.

Bieling, P., Hansen, S. D., Akin, O., Li, T.-D., Hayden, C. C., Fletcher, D. A., et al. (2018). WH2 and proline-rich domains of WASP-family proteins collaborate to accelerate actin filament elongation. *EMBO J.* 37, 102–121. doi:10.15252/embj.201797039.

Boukhelifa, M., Moza, M., Johansson, T., Rachlin, A., Parast, M., Huttelmaier, S., et al. (2006). The proline-rich protein palladin is a binding partner for profilin. *FEBS J.* 273, 26–33. doi:10.1111/j.1742-4658.2005.05036.x.

Camera, P., da Silva, J. S., Griffiths, G., Giuffrida, M. G., Ferrara, L., Schubert, V., et al. (2003). Citron-N is a neuronal Rho-associated protein involved in Golgi organization through actin cytoskeleton regulation. *Nat. Cell Biol.* 5, 1071–1078. doi:10.1038/ncb1064.

Courtemanche, N. (2018). Mechanisms of formin-mediated actin assembly and dynamics. *Biophys. Rev.* 10, 1553–1569. doi:10.1007/s12551-018-0468-6.

Gareus, R., Di Nardo, A., Rybin, V., and Witke, W. (2006). Mouse profilin 2 regulates endocytosis and competes with SH3 ligand binding to dynamin 1. *J. Biol. Chem.* 281, 2803–2811. doi:10.1074/jbc.M503528200.

Gertler, F. B., Niebuhr, K., Reinhard, M., Wehland, J., and Soriano, P. (1996). Mena, a relative of VASP and Drosophila Enabled, is implicated in the control of microfilament dynamics. *Cell* 87, 227–239. doi:10.1016/s0092-8674(00)81341-0.

Giesemann, T., Rathke-Hartlieb, S., Rothkegel, M., Bartsch, J. W., Buchmeier, S., Jockusch, B. M., et al. (1999). A role for polyproline motifs in the spinal muscular atrophy protein SMN. Profilins bind to and colocalize with smn in nuclear gems. *J. Biol. Chem.* 274, 37908–37914. doi:10.1074/jbc.274.53.37908.

Giesemann, T., Schwarz, G., Nawrotzki, R., Berhörster, K., Rothkegel, M., Schlüter, K., et al. (2003). Complex formation between the postsynaptic scaffolding protein gephyrin, profilin, and Mena: a possible link to the microfilament system. *J. Neurosci. Off. J. Soc. Neurosci.* 23, 8330–8339.

Huang, R., Yuan, D.-J., Li, S., Liang, X.-S., Gao, Y., Lan, X.-Y., et al. (2020). NCAM regulates temporal specification of neural progenitor cells via profilin2 during corticogenesis. *J. Cell Biol.* 219. doi:10.1083/jcb.201902164.

Lafuente, E. M., van Puijenbroek, A. A. F. L., Krause, M., Carman, C. V., Freeman, G. J., Berezovskaya, A., et al. (2004). RIAM, an Ena/VASP and Profilin ligand, interacts with Rap1-GTP and mediates Rap1-induced adhesion. *Dev. Cell* 7, 585–595. doi:10.1016/j.devcel.2004.07.021.

Lambrechts, A., Kwiatkowski, A. V., Lanier, L. M., Bear, J. E., Vandekerckhove, J., Ampe, C., et al. (2000). cAMP-dependent protein kinase phosphorylation of EVL, a Mena/VASP relative, regulates its interaction with actin and SH3 domains. *J. Biol. Chem.* 275, 36143–36151. doi:10.1074/jbc.M006274200.

Lederer, M., Jockusch, B. M., and Rothkegel, M. (2005). Profilin regulates the activity of p42POP, a novel Myb-related transcription factor. *J. Cell Sci.* 118, 331–341. doi:10.1242/jcs.01618.

Mammoto, A., Sasaki, T., Asakura, T., Hotta, I., Imamura, H., Takahashi, K., et al. (1998). Interactions of drebrin and gephyrin with profilin. *Biochem. Biophys. Res. Commun.* 243, 86–89. doi:10.1006/bbrc.1997.8068.

Mondin, M., Carta, M., Normand, E., Mulle, C., and Coussen, F. (2010). Profilin II regulates the exocytosis of kainate glutamate receptors. *J. Biol. Chem.* 285, 40060–40071. doi:10.1074/jbc.M110.140442.

Mouneimne, G., Hansen, S. D., Selfors, L. M., Petrak, L., Hickey, M. M., Gallegos, L. L., et al. (2012). Differential remodeling of actin cytoskeleton architecture by profilin isoforms leads to distinct effects on cell migration and invasion. *Cancer Cell* 22, 615–630. doi:10.1016/j.ccr.2012.09.027.

Ohta, K., Matsumoto, Y., and Nishio, M. (2019). Profilin2 is required for filamentous actin formation induced by human parainfluenza virus type 2. *Virology* 533, 108–114. doi:10.1016/j.virol.2019.05.013.

Rebowski, G., Boczkowska, M., Drazic, A., Ree, R., Goris, M., Arnesen, T., et al. (2020). Mechanism of actin N-terminal acetylation. *Sci. Adv.* 6, eaay8793. doi:10.1126/sciadv.aay8793.

Ree, R., Kind, L., Kaziales, A., Varland, S., Dai, M., Richter, K., et al. (2020). PFN2 and NAA80 cooperate to efficiently acetylate the N-terminus of actin. *J. Biol. Chem.* 295, 16713–16731. doi:10.1074/jbc.RA120.015468.

Reinhard, M., Giehl, K., Abel, K., Haffner, C., Jarchau, T., Hoppe, V., et al. (1995). The proline-rich focal adhesion and microfilament protein VASP is a ligand for profilins. *EMBO J.* 14, 1583–1589.

Shao, J., Welch, W. J., Diprospero, N. A., and Diamond, M. I. (2008). Phosphorylation of profilin by ROCK1 regulates polyglutamine aggregation. *Mol. Cell. Biol.* 28, 5196–5208. doi:10.1128/MCB.00079-08.

Sharma, A., Lambrechts, A., Hao, L. T., Le, T. T., Sewry, C. A., Ampe, C., et al. (2005). A role for complexes of survival of motor neurons (SMN) protein with gemins and profilin in neurite-like cytoplasmic extensions of cultured nerve cells. *Exp. Cell Res.* 309, 185–197. doi:10.1016/j.yexcr.2005.05.014.

Skare, P., Kreivi, J.-P., Bergström, A., and Karlsson, R. (2003). Profilin I colocalizes with speckles and Cajal bodies: a possible role in pre-mRNA splicing. *Exp. Cell Res.* 286, 12–21. doi:10.1016/s0014-4827(03)00102-2.

Stüven, T., Hartmann, E., and Görlich, D. (2003). Exportin 6: a novel nuclear export receptor that is specific for profilin.actin complexes. *EMBO J.* 22, 5928–5940. doi:10.1093/emboj/cdg565.

Suetsugu, S., Miki, H., and Takenawa, T. (1998). The essential role of profilin in the assembly of actin for microspike formation. *EMBO J.* 17, 6516–6526. doi:10.1093/emboj/17.22.6516.

Tanaka, Y., Nonaka, T., Suzuki, G., Kametani, F., and Hasegawa, M. (2016). Gain-of-function profilin 1 mutations linked to familial amyotrophic lateral sclerosis cause seed-dependent intracellular TDP-43 aggregation. *Hum. Mol. Genet.* 25, 1420–1433. doi:10.1093/hmg/ddw024.

Wang, R., Cleary, R. A., Wang, T., Li, J., and Tang, D. D. (2014). The association of cortactin with profilin-1 is critical for smooth muscle contraction. *J. Biol. Chem.* 289, 14157–14169. doi:10.1074/jbc.M114.548099.

Wang, X., Kibschull, M., Laue, M. M., Lichte, B., Petrasch-Parwez, E., and Kilimann, M. W. (1999). Aczonin, a 550-kD putative scaffolding protein of presynaptic active zones, shares homology regions with Rim and Bassoon and binds profilin. *J. Cell Biol.* 147, 151–162. doi:10.1083/jcb.147.1.151.

Witke, W., Podtelejnikov, A. V., Di Nardo, A., Sutherland, J. D., Gurniak, C. B., Dotti, C., et al. (1998). In mouse brain profilin I and profilin II associate with regulators of the endocytic pathway and actin assembly. *EMBO J.* 17, 967–976. doi:10.1093/emboj/17.4.967.

Wüstenhagen, E., Boukhallouk, F., Negwer, I., Rajalingam, K., Stubenrauch, F., and Florin, L. (2018). The Myb-related protein MYPOP is a novel intrinsic host restriction factor of oncogenic human papillomaviruses. *Oncogene* 37, 6275–6284. doi:10.1038/s41388-018-0398-6.

Zaidi, A. H., and Manna, S. K. (2016). Profilin-PTEN interaction suppresses NF-κB activation via inhibition of IKK phosphorylation. *Biochem. J.* 473, 859–872. doi:10.1042/BJ20150624.

Zhao, W. M., Jiang, C., Kroll, T. T., and Huber, P. W. (2001). A proline-rich protein binds to the localization element of Xenopus Vg1 mRNA and to ligands involved in actin polymerization. *EMBO J.* 20, 2315–2325. doi:10.1093/emboj/20.9.2315.
